# Corona phobia and mental health among nurses: identifying determinants in a cross-sectional survey

**DOI:** 10.3389/fpubh.2025.1681478

**Published:** 2025-10-30

**Authors:** Menevse Yildirim, Emre Yildiz, Seyda Seren Intepeler

**Affiliations:** ^1^Department of Nursing, Fethiye Faculty of Health Sciences, Muğla Sıtkı Koçman University, Muğla, Türkiye; ^2^Dokuz Eylul University Hospital, Izmir, Türkiye; ^3^Dokuz Eylul University, Faculty of Nursing, Izmir, Türkiye; ^4^Dokuz Eylul Universitesi Hemsirelik Fakultesi, Izmir, Türkiye

**Keywords:** COVID-19, corona phobia, nurses, mental health, pandemic, well-being

## Abstract

**Background:**

Assessing the psychological impact of the pandemic on nurses is essential for protecting their well-being and ensuring the resilience of healthcare systems.

**Methods:**

A descriptive, cross-sectional study following the STROBE reporting guidelines. The study included 417 nurses from Dokuz Eylul University Research and Practice Hospital who participated voluntarily. Data were collected between July and October 2021 using the Sociodemographic Data Form, the COVID-19 Phobia Scale (C19P-S), and the Mental Health Continuum Short Form (MHC-SF). Descriptive statistics and multiple linear regression analysis were used (*p* < 0.05).

**Results:**

The mean C19P-S score was 49.03 ± 17.29. Gender, education, perceived general health status, and intention to quit predicted 17% of COVID-19 phobia variance (*R*^2^ = 0.17, *p* < 0.05). The mean MHC-SF score was 34.31 ± 16.53. Categorically, 46.8% of nurses were languishing in the emotional subdimension, 42.4% were mentally healthy, and 25% were languishing in social and psychological well-being.

**Conclusion:**

Nurses experienced moderate COVID-19 phobia, with female gender, undergraduate education, worse perceived health, and intention to quit emerging as significant predictors, collectively explaining 17% of the variance. Interventions are needed to strengthen nurses’ mental health, particularly emotional well-being. Healthcare policymakers and administrators should implement strategies to support nurses’ psychological well-being and foster a fear-free work environment through empowerment.

## Highlights

- Nurses experienced a high level of COVID-19 phobia, with greater psychological and social impacts compared to somatic and economic effects.
- Nearly half of the nurses exhibited emotional languishing, while a quarter experienced psychological and social languishing, reflecting the substantial mental health burden they faced.
- Sex, education, perceived general health status, and intention to quit collectively accounted for 17% of the variance in COVID-19 phobia.

## Introduction

The novel virus SARS-CoV-2 first emerged in Wuhan, China, in late 2019. Its rapid global spread led the World Health Organization (WHO) to declare a pandemic on March 11, 2020 (1). Since its emergence, the virus has caused significant morbidity and mortality worldwide. As of January 31, 2024, a total of 774,291,287 cases and 7,019,704 deaths have been reported globally (2), while in Turkey, the Ministry of Health recorded 17,232,066 cases and 102,174 deaths as of March 2023 (3).

While the immediate concern during the outbreak focused on physical health outcomes, mounting evidence suggests that the pandemic also exerted profound psychological and social effects across populations (4–6). Among healthcare workers (HCWs) at the frontline, these effects are particularly pronounced. The combination of rapid viral transmission, high fatality rates, and systemic healthcare pressures has led to a novel psychological response, termed “corona phobia,” which reflects excessive fear and anxiety related to COVID-19 (7–9). This construct aligns with the DSM-IV criteria for specific phobias and has been validated using tools such as the COVID-19 Phobia Scale (9). Prior studies indicate that corona phobia is associated with anxiety, depression, distress, and sleep disturbances among both healthcare professionals and the general population, highlighting its relevance for mental health interventions (10–12).

Healthcare professionals have been disproportionately impacted by the dual physical and psychological stresses associated with the pandemic. WHO estimated that 35 million healthcare workers had been infected globally (1). According to the International Council of Nurses (ICN), more than 20,000 healthcare workers lost their lives during the pandemic, including 1,500 nurses across 44 countries (13). In Turkey, as of April 29, 2020, 7,428 HCWs had contracted the virus, accounting for 6.5% of all reported cases at that time (14).

The psychological burden for HCWs extends beyond the fear of infection and mortality. Nurses, in particular, have reported high levels of anxiety, depression, burnout, and moral distress due to their exposure to critically ill patients, ethical dilemmas in triage decisions, and the risk of transmitting the virus to their families (10, 11, 15). Research indicates that corona phobia significantly influences professional turnover intention (*β* = 0.316) and organizational turnover intention (*β* = 0.424) among HCWs, further straining healthcare systems (16). These challenges were further compounded by the pressures of equitably allocating limited resources, balancing personal and professional health needs, and addressing the demands of an overstretched healthcare system (17). Furthermore, factors such as social support, institutional resources, access to personal protective equipment (PPE), resilience, and media exposure may modulate the psychological impact of the pandemic, suggesting that additional unmeasured variables contribute to mental health outcomes (12, 18–20).

While numerous studies have explored the impact of the pandemic on different populations (21–26), research specifically examining corona phobia among nurses and its determinants remains scarce. Understanding these psychological burdens is essential for developing targeted interventions that enhance nurses’ mental well-being and resilience. Addressing these challenges may not only enhance nurses’ well-being but also strengthen healthcare systems’ preparedness and effectiveness in future public health crises. Accordingly, this study aimed to examine Coronavirus Phobia (COVID-19) and mental health conditions among nurses and to identify factors predicting COVID-19 phobia.

## Methods

### Study design

A descriptive, cross-sectional study following the STROBE reporting guidelines.

### Setting and participants

The study included 417 nurses from Dokuz Eylul University Research and Practice Hospital. Using 95% confidence level, alpha equals 0.05, known value (Mean = 40.00), mean of the population (34.51), the standard deviation of the sampled population (16.53) and sample size 417, the power of the study was calculated, and determined to be 100% (27, 28).

### Data sources/measurement

The data collection instruments were distributed to all nurses via Google Forms. Preliminary information about the study was provided electronically, and data were collected from nurses who voluntarily agreed to participate with system approval. Online self-report questionnaires were preferred, as they are widely recommended in psychological and mental health research for capturing subjective perceptions and emotional states that cannot be directly observed. Studies conducted with healthcare workers during the COVID-19 pandemic have reported that online self-report methods are both safe and feasible, minimizing face-to-face contact, and facilitating the data collection process (29–31). After the data collection, participants were informed that the study results would be shared with them after publication, and the research team’s contact information was provided for any inquiries. Additionally, the findings, results, and recommendations obtained from the study were shared with the hospital’s nursing services management.

#### The sociodemographic and job characteristics data form

The form delineates the participants’ sociodemographic and professional characteristics. This scale consists of 17 questions, including information such as age, sex, educational status, marital status, working status in pandemic clinics, working time in pandemic clinics, working time in the institution, working time in the profession, and average number of patients cared for during the pandemic.

#### COVID-19 phobia scale (C19P-S)

The development of the scale was motivated by the necessity to assess the potential for the emergence of new phobias in response to the pandemic. This scale was developed to measure the potential development of phobia related to the novel coronavirus, and its validity and reliability have been established. The scale utilizes a five-point Likert scale, ranging from 1 (strongly disagree) to 5 (strongly agree). The scale is composed of four subdimensions and 20 items. The psychological subdimension is measured by the first five items, the somatic subdimension by the sixth and twelfth items, the social subdimension by the seventh, eleventh, and sixteenth items, and the economic subdimension by the eighth, fourteenth, and sixteenth items. The subdimension scores are derived by summing the scores of the responses to the items that fall within a given subdimension. The total C19P-S score of the scale is determined by aggregating the subdimension scores. The scale score ranges from 20 to 100 points. Higher scores indicate greater levels of coronaphobia in subdimensions and overall. It is noteworthy that the scale does not include reverse-scored items, and the Cronbach’s alpha value is reported as 0.92 (9). In the present study, Cronbach’s alpha was determined to be 0.95, suggesting a high degree of internal consistency in the scale’s measurements.

#### The mental health continuum short form (RSS-CF)

The scale in question is a self-report scale developed by Keyes et al. (32) that measures emotional, social, and psychological well-being characteristics. It represents the mental health continuum. The self-report component of the scale is derived from the individual’s self-reported experiences and perceptions. The validity and reliability of the scale were established by Demirci and Akin (33). It consists of 14 items and three subdimensions: emotional (items 1, 2, and 3); social well-being (items 4, 5, 6, 7, and 8); and psychological (items 9, 10, 11, 12, 13, and 14). The scale utilizes a 6-point Likert scale, ranging from 0, representing “Never,” to 5, representing “Every day.” The total score that can be obtained from the scale varies between 0 and 70. It is noteworthy that the scale does not include reverse-scored items. The total score pertaining to mental health continuity was derived by summing the 14 items that comprise the scale. Additionally, the subscales can be scored independently. High scores on each subscale are indicative of high well-being in that specific area. Individuals who indicated “almost every day” or “every day” in response to one of the three statements in the emotional well-being dimension of the scale, and those who selected “almost every day” or “every day” in 6 of the 11 statements in the psychological and social well-being dimension, were classified as having good well-being. Conversely, individuals who selected “never” or “once or twice” in one of the three statements in the emotionality dimension of the scale, and those who selected “never” or “once or twice” in 6 of the 11 statements in the psychological and social well-being dimension, were categorized as having poor well-being. Other states indicate normal mental health. The reliability coefficients of the scale were found to be 0.84, 0.78, and 0.85 for the three subscales and 0.90 for the entire scale (34). In the present study, Cronbach’s alpha was determined to be 0.94.

### Data analyses

The collected data were then subjected to analysis using SPSS version 25.0. Given that the research data were collected online, with respondents being required to complete each question before advancing to the next, no missing data were observed. The presence of outliers was assessed through the utilization of box plots, a method of graphical representation. This analysis yielded no identification of extreme values. Descriptive statistics, encompassing frequency, percentage, mean, standard deviation, minimum, median, and maximum, were employed to summarize the data. The normality of the data distribution was assessed using Shao’s skewness and kurtosis criteria, and parametric tests were applied to variables that satisfied the normality assumption.

For the purpose of group comparisons, independent samples *t*-tests were employed to compare two groups, while analysis of variance (ANOVA) was utilized for comparisons involving three or more independent groups. When significant differences between groups were identified, Bonferroni *post hoc* analysis was conducted to determine which specific pairs of groups differed significantly. Pearson correlation analysis was performed to examine the relationship between C19P-S and the independent variables. Furthermore, multiple linear regression analysis was executed for multivariate analysis using both the “enter” and “stepwise” methods. The residuals were confirmed to follow a normal distribution, linearity was assessed through residual vs. fitted plots, and homoscedasticity was evaluated through visual inspection of residual plots. The multicollinearity assumption was tested, and no issues were detected. A significance level of 0.05 was established for all statistical analyses.

### Ethics approval and consent to participate

To conduct the study, we obtained legal permission from the Ministry of Health, Dokuz Eylul University Research and Practice Hospital, and Dokuz Eylül University Non-Interventional Research Ethics Committee (Date: 15.03.2021, Decision Number: 2021/09-10), as well as from the scale owners. Nurses who volunteered to participate in the study provided informed consent through the Google Forms system.

## Results

### Descriptive data

The sociodemographic and job characteristics of the nurses during the pandemic are presented in Table 1.

**Table 1:** Sociodemographic and job characteristics of nurses (*n* = 417).

Characteristics	Category	*n*	%
Age ( $\bar{x}$ = 33.75 ± 7.94)	20–30 years	183	43.9
	31–40 years	129	30.9
	≥41 years	105	25.2
Sex	Male	39	9.4
	Woman	378	90.6
Education	High school and associate degree	7	1.7
	License	356	85.4
	Postgraduate	54	12.9
Marital status	Married	234	56.1
	Single	183	43.9
Duration of working at the institution ( $\bar{x}$ = 9.29 ± 8.43)	≤10 years	244	58.5
	11–20 years	114	27.3
	≥21 years	59	14.1
Duration of working in the profession ( $\bar{x}$ = 10.58 ± 8.50)	≤10 years	221	53.0
	11–20 years	127	30.5
	≥21 years	69	16.5
Working status in pandemic units	Yes	244	58.5
	No	173	41.5
The duration of employment in pandemic outpatient clinics ( $\bar{x}$ = 0.48 ± 2.52)			
The duration of employment in pandemic services ( $\bar{x}$ = 1.52 ± 3.32)			
The duration of employment in pandemic intensive care units ( $\bar{x}$ = 1.69 ± 4.51)			
Patients cared for in a shift before the pandemic ( $\bar{x}$ = 26.45 ± 82.85)	≤10 patient	221	53.0
	11–20 patient	127	30.5
	≥21 patient	69	16.5
Patients cared for in one shift during the pandemic ( $\bar{x}$ = 14.37 ± 33.65)	≤10 patient	330	79.1
	11–20 patient	47	11.3
	≥21 patient	40	9.6
Number of family members living in the same house (including you)	1	84	20.1
	2	85	20.4
	3 or more	248	59.5
Shift	Only during the day	93	22.3
	Day and night	321	77.0
	Only at night	3	0.7
COVID (+) diagnosis	Yes	99	23.7
	No	318	76.3
Experiencing isolation due to high risk	Yes	158	37.9
	No	259	62.1
Intention to quit before the pandemic	Yes	75	18.0
	No	342	82.0
Intention to quit during the pandemic	Yes	98	23.5
	No	319	76.5
Perceived general health status compared to before the pandemic	Much better	9	2.2
	Better	41	9.8
	Same	216	51.8
	Worse	135	32.4
	Much worse	16	3.8

The mean age of the nurses participating in the study was 33.75 years (±7.94 years), with a range from 22 to 55 years. The majority of the nurses were female (90.6%), had a university degree (85.4%), and were married (56.1%). The average duration of employment at the institution was 9.29 years (±8.43), and the average duration of employment in the profession was 10.58 years (±8.50). The majority of the participants (58.5%) were employed in pandemic units. The average duration of nurses working in pandemic outpatient clinics was 0.48 ± 2.52 months, the average duration of nurses working in pandemic intensive care units was 1.52 ± 3.32 months, and the average duration of nurses working in pandemic services was 1.69 ± 4.51 months. The mean number of patients cared for during a shift before the pandemic was 26.45 ± 82.85, while the mean number of patients cared for during a shift during the pandemic was 14.37 ± 33.65. It was determined that more than half of the participants resided with three or more individuals (maximum: 6, mean: 2.77 ± 1.25, 59.5%). Furthermore, 77% of the participants reported working both day and night shifts. The majority (76.3%) of these participants had been diagnosed with COVID-19, and the majority of them were isolated due to high-risk contact (62.1%). Moreover, 82% of nurses had previously expressed their intention to resign before the pandemic, and 76.5% indicated a similar intention during the pandemic. Furthermore, the data indicates that more than half of the nurses reported no change in their general health status before and during the pandemic, while more than one-third indicated a deterioration in their health (36.2%) (Table 1).

The findings related to the evaluation of COVID-19 phobia and mental health among the nurses participating in the study are presented in Table 2.

**Table 2:** Scores on the C19P-S and MHC-SF scales and subdimensions (*n* = 417).

Scales and subdimentions	Minimum	Maximum	Median	Mean ± Std. deviation
C19P-S	20.00	100.00	47.00	49.03 ± 17.29
Psychological	6.00	31.00	17.00	17.75 ± 6.03
Somatic	5.00	25.00	10.00	9.98 ± 4.60
Social	5.00	25.00	13.00	12.89 ± 4.92
Economic	4.00	20.00	8.00	8.41 ± 3.60
MHC-SF	0.00	70.00	35.00	34.31 ± 16.53
Emotional	0.00	15.00	5.00	5.67 ± 3.96
Social well-being	0.00	25.00	11.00	11.32 ± 6.43
Psychological	0.00	30.00	18.00	17.31 ± 8.23

The mean C19P-S score of the nurses was 49.03 ± 17.29, and their mean MHC-SF score was 34.31 ± 16.53. The nurses demonstrated average scores of 17.75 ± 6.03 in the psychological subdimension of the C19P-S, 9.98 ± 4.60 in the somatic subdimension, 12.89 ± 4.92 in the social subdimension, and 8.41 ± 3.60 in the economic subdimension (Table 2).

When the MHC-SF subdimensions were categorized and examined, nearly half of the nurses (46.8%) experienced languishing in the “emotional” subdimension. According to the subdimensions of “social well-being” and “psychological” dimensions, 42.4% of the nurses exhibited normal mental health, while 25% were classified as languishing.

Multiple regression analysis was conducted using the enter method to assess the impact of 17 independent variables, mental health continuity, and subdimension scores on the C19P-S scores of the nurses. While a statistically significant model was established (*p* < 0.05; *F* = 4.841; Durbin–Watson = 1.782), the regression analysis was repeated using the stepwise method to ensure that significant variables remained in the model and to address the issue of multicollinearity (VIF > 10). The final model revealed that factors such as sex, educational attainment, perception of general health status before the pandemic, and the decision to discontinue employment during the pandemic collectively accounted for 17% of the variance in the manifestation of fear associated with the novel virus. However, it is noteworthy that for 83% of the participants, the incidence of the virus was influenced by other variables (see Table 3).

**Table 3:** Factors predicting nurses’ coronavirus-19 phobia (final model).

Model	*B*	Std. error	β	*t*	*p*	95% confidence interval for *β*		Collinearity statistics	
						Lower bound	Upper bound	Tolerance	VIF
Constant	27.686	3.583	–	7.726	0.000^*^	20.641	34.730	–	–
Sex (male)	10.825	2.686	0.182	4.030	0.000^*^	5.545	16.106	0.976	1.025
Education (postgraduate)	5.899	2.325	0.121	2.537	0.012^*^	1.328	10.469	0.884	1.131
Perceived health: worse (much better)	8.470	1.775	0.229	4.772	0.000^*^	4.981	11.960	0.865	1.156
Perceived health: much worse (much better)	16.412	4.165	0.182	3.941	0.000^*^	8.225	24.598	0.933	1.072
Intention to quit (no)	6.913	1.913	0.170	3.614	0.000^*^	3.153	10.673	0.908	1.102

^†^*N* = 417, *R*^2^ = 0.176, Adjusted *R*^2^ = 0.166, *F* = 17.567, ^*^p = 0.000, Durbin–Watson = 1.829, VIF<10.

The mean C19P-S score of female nurses was 10.825 points higher than that of male nurses. Furthermore, nurses with an undergraduate education exhibited a mean C19P-S score that was 5.899 points higher than nurses with a graduate education. In addition, nurses with “worse” and “much worse” health statuses before the pandemic exhibited mean C19P-S scores that were 8.470 and 16.412 points higher, respectively, compared to those with “much better” health status. Lastly, nurses who expressed intentions to resign during the pandemic exhibited a mean C19P-S score that was 6.913 points higher compared to those who did not intend to resign (see Table 3).

## Discussion

Nurses working during the COVID-19 pandemic have faced various psychological challenges, including fear, stress, anxiety, and mental health issues (10, 11, 15, 16). These difficulties impact the well-being of healthcare professionals and have also posed considerable challenges to the effective management of patient care and workforce planning (34). Moreover, understanding these challenges is crucial for informing interventions that support nurses’ psychological well-being.

This study explores nurses’ experiences of fear and mental health during the pandemic, as well as the factors predicting COVID-19-related fear. A study by Gholampour et al. (35) revealed that the prevalence of COVID-19 phobia varied across studies and was particularly high among healthcare professionals, especially nurses. Similarly, a study conducted in Saudi Arabia reported that 80.3% of nurses experienced COVID-19 phobia (36). Fronda and Labrague (16) also found that more than half of nurses exhibited symptoms of COVID-19 phobia.

In the present study, nurses were found to have a high level of COVID-19 phobia. While psychological and social subdimension scores were elevated, moderate levels of corona phobia were observed in the somatic and economic subdimensions. Two studies conducted in Turkey with intensive care nurses and nursing students yielded similar findings regarding total Coronaphobia Scale scores and subdimension means (21, 37). These results suggest that while nurses are affected across all dimensions, psychological and social impacts are particularly pronounced. Being on the front lines, constantly dealing with social distancing measures, and facing “stay-at-home” restrictions may have contributed to these findings. Korkut (38) also demonstrated that healthcare workers exhibited heightened levels of corona phobia. Although the prevalence of COVID-19 phobia among nurses has been well-documented in the literature, it is possible that phobic reactions may intensify during epidemics. Contributing factors include direct contact with COVID-19 patients, limited access to PPE, quarantine conditions, feelings of hopelessness, and burnout (36, 38–40).

The findings of this study indicated that nurses’ mental health was at a moderate level. A significant proportion exhibited symptoms of emotional languishing, while a notable segment also experienced psychological and social languishing. These results align with those of a study conducted with intensive care nurses in Turkey (37). Similarly, a study in Italy reported that healthcare workers had moderate mental health, though a smaller proportion (8.9%) experienced mental languishing (28). Differences in epidemic management strategies across countries may have influenced these variations. The existing literature frequently examines stress, depression, anxiety, and burnout as key indicators of mental health (41, 42). A national study in the United States found that the vast majority of nurses (84.7%) experienced moderate burnout, while nearly half exhibited moderate to severe symptoms of depression (44.6%) and post-traumatic stress (46.7%) (39). Strengthening resilience and enhancing mentalizing capacity among healthcare workers have been recognized as crucial in mitigating the adverse psychological effects of the pandemic, including anxiety, stress, and depression (43). Taken together, these findings emphasize the importance of supporting nurses’ mental health through targeted interventions and institutional policies.

In the context of caring for COVID-19 patients, factors such as life-threatening situations, separation from home, quarantine practices, and ethically challenging decisions have been shown to significantly impact nurses’ mental health (39, 41). However, the extent of these effects varies depending on factors such as geographical location, sample size, institutional framework, and working conditions.

To the best of our knowledge, very few studies have specifically examined the predictors of corona phobia among nurses (44, 45). In this study, sex, educational status, perceived general health status before the pandemic, and intention to quit were significant predictors of corona phobia, collectively explaining 17% of the variance. While the regression model is a major strength, the low explained variance represents a limitation that must be acknowledged. A substantial proportion of the variance (83%) remains unexplained, suggesting the role of additional unmeasured factors. Potential contributors include institutional support, access to PPE, pre-existing mental health conditions, social support outside work, individual resilience, and media exposure (12, 18–20, 46).

Evidence from the literature further supports the psychological impact of COVID-19 fear. A meta-analysis by Şimşir et al. (46) reported strong associations between fear of COVID-19 and anxiety, traumatic stress, and distress, as well as moderate associations with stress and depression in the general population. Lin et al. (12) found that social media use and misunderstanding of COVID-19 were linked to increased psychological distress. Yıldırım and Güler (18) highlighted that perceived health, self-efficacy, and preventive behaviors influenced mental health outcomes among Turkish healthcare workers. Eder et al. (19) demonstrated that individual and environmental factors predicted fear and perceived health across countries, and Balaban and Potas (20) showed that fear of illness, virus evaluation, and quality of life significantly affected social anxiety and fear in patients with chronic conditions.

A separate study identified gender, marital status, job status, and personal resilience as significant predictors of corona phobia, accounting for 15% of the variance (44). Additionally, previous research has highlighted hygiene habits as a key factor influencing COVID-19 phobia (47) Similarly, a study conducted with nursing students found that factors such as experiencing symptoms of the virus, the loss of a relative or acquaintance due to COVID-19, fear of caring for infected patients, lack of social distancing, and shared living spaces (e.g., toilets, elevators) contributed to corona phobia, explaining 35% of the variance (21). Furthermore, while some studies suggest that women tend to experience higher levels of fear compared to men (46), others indicate that sex is not a significant differentiating factor (35). Collectively, these findings underscore that nurses’ corona phobia is a multidimensional phenomenon shaped by individual, occupational, and environmental determinants. It is hypothesized that higher education levels foster professionalism and better information processing, which may aid in managing COVID-19-related fears. Nurses who perceive their health status as poor may experience heightened concern about their future well-being. Nurses contemplating resignation may experience heightened corona phobia, possibly due to exposure to patient mortality or the loss of colleagues, further amplified by extensive media coverage.

### Strengths and limitations of the study

This study offers significant insights into the prevalence of fear surrounding the COVID-19 and the mental health status of nurses, underscoring the profound impact of the pandemic on healthcare professionals who play a pivotal role in public health maintenance. As with the general population, nurses have experienced a variety of fears during the pandemic; however, their role imposes unique demands on their mental and emotional well-being. While the study identifies predictive factors contributing to the development of fear of the novel virus, the use of nonprobability sampling may introduce biases that limit the findings’ generalizability. A comprehensive understanding of corona phobia necessitates the exploration of potential confounding factors, including the workplace environment and access to mental health resources. Future research endeavors should prioritize the incorporation of larger samples and the implementation of confounder control mechanisms to enhance the robustness of the findings. It is incumbent upon policymakers to devise targeted interventions to address epidemic-specific phobias, thereby promoting the mental health and well-being of nurses and ensuring their capacity to deliver essential care during public health crises.

## Conclusion

This study underscores the significant impact of COVID-19-related fear and anxiety on nurses’ psychological well-being. Nurses who perceive their health as poor and those contemplating resignation were identified as particularly vulnerable to heightened levels of COVID-19-related fear. Based on the predictor variables identified in this study—female gender, undergraduate education, poorer perceived general health, and intention to quit—there is a clear need for targeted interventions tailored to at-risk groups.

Specifically, high-risk nurses should have access to structured psychological support programs, such as cognitive-behavioral therapy and resilience training (12, 18). Priority should be given to female nurses and those with perceived poor health. For nurses considering resignation during the pandemic, retention-focused strategies addressing pandemic-specific concerns—such as provision of mental health support, flexible scheduling, access to adequate personal protective equipment, and mentoring opportunities—are essential (19, 47). Moreover, healthcare organizations should cultivate empowering work environments characterized by transparent communication, sufficient resource allocation, and readily accessible mental health services. Complementary educational initiatives and structured social support mechanisms can further enhance nurses’ coping abilities, resilience, and overall well-being.

The integrated implementation of these measures is expected to mitigate COVID-19-related fear, strengthen nurses’ psychological well-being, and improve both nursing and patient care outcomes. This evidence-based approach provides a comprehensive framework for policymakers and healthcare administrators to guide interventions during the current pandemic as well as in future public health crises.

## Data Availability

The raw data supporting the conclusions of this article will be made available by the authors, without undue reservation.
